# Editorial: Innovations in older adult care and health service management: a focus on the Asia-Pacific region

**DOI:** 10.3389/fpubh.2024.1369827

**Published:** 2024-01-31

**Authors:** Madhan Balasubramanian, Angie A. Shafei, Zhanming Liang

**Affiliations:** ^1^Flinders University, College of Business Government and Law, Healthcare Management and Centre for Social Impact, Adelaide, SA, Australia; ^2^The University of Sydney, Faculty of Medicine and Health, School of Public Health, Menzies Centre for Health Policy and Economics, Sydney, NSW, Australia; ^3^The University of Adelaide, Faculty of Health Sciences, Australian Research Centre for Population Oral Health, Adelaide, SA, Australia; ^4^Flinders University, College of Business Government and Law, Healthcare Management, Adelaide, SA, Australia; ^5^James Cook University, College of Public Health, Medical and Vet Sciences, Australian Institute of Tropical Health and Medicine, Townsville, QLD, Australia

**Keywords:** older adults, aged care, Asia-Pacific, health workforce, models of care, health systems

## Introduction

Population aging in the Asia-Pacific Region is a significant challenge for the 21st century. With countries in the Asia-Pacific aging faster than any region in the world, demographic transition and challenges associated with it have grown to utmost social, political, healthcare, and economic significance. Nearly 60 percent of the world's population over 60 years of age reside in the Asia-Pacific region, which amounts to 630 million people (1). This population is projected to reach 1.3 billion by 2050 (1). In general, older adults exhibit higher prevalence of chronic conditions, comorbidities, and hospital admissions, which in turn elevate health care costs. Women comprise of over 60% of the older adult population in the Asia Pacific (2). Gender imbalance has implications for social support and health care systems, given that women typically have greater life expectancy and can face more years of potential disability. Given the traditional role of women as family caregivers in many societies in the region, older women could face greater pressure potentially impacting their health and wellbeing. Traditional models of health workforce development and service delivery are less likely to be effective in addressing the growing concerns if the focus remains centered on specialized and tertiary care. It is essential to strengthen primary and community-based models of care that caters to the growing needs of and are sensitive to cultural contexts, local challenges, and specific requirements of older adults.

**Figure 1:**
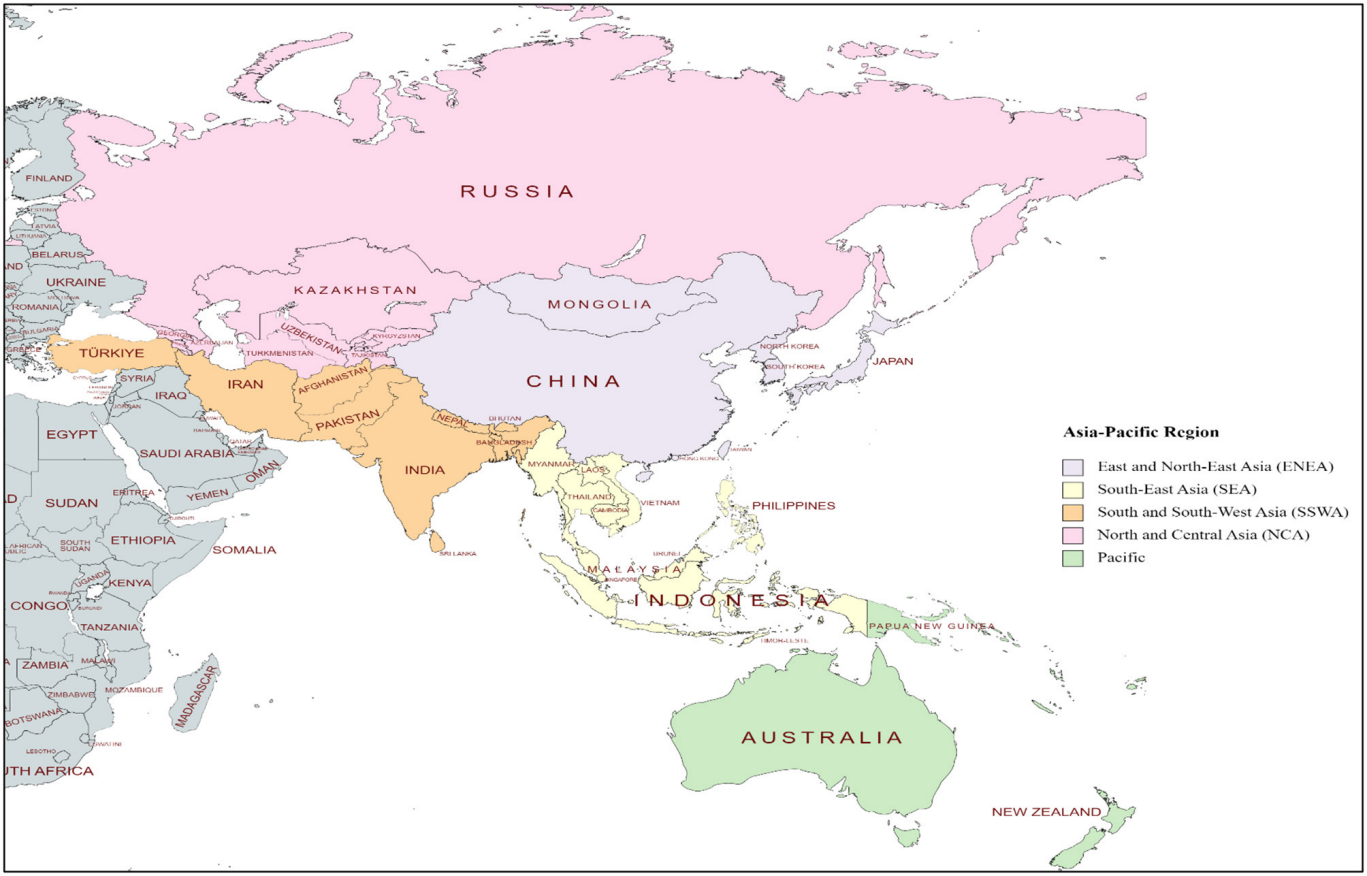
The Asia-Pacific Region. Asia-Pacific subregions and country classification is based on the United Nations Economic and Social Commission for Asia and the Pacific categorization. This map has been generated utilizing mapping tools available at mapchart.net, licensed under CC BY-SA 4.0.

Based on the United Nations Economic and Social Commission of Asia and the Pacific, the region is divided into five divisions (1): (i) East and Northeast Asia, (ii) South-East Asia, (iii) South and South-West Asia, (iv) North and Central Asia, and (v) Pacific (see Figure 1). The 56 countries in this region come with varying levels of social and economic progress. The World Bank identifies two of these countries as low-income economies, 26 as lower middle-income economies, 17 as upper middle-income economies and 11 as high-income economies (see Supplementary Table 1). Over the last two decades, China, India, Indonesia, Philippines, Thailand, and Vietnam consistently outperformed other emerging markets in the world economically. In addition to the fast-paced economic growth in the region, Asia-Pacific countries share a rich and diverse cultural history. In many cultures, particularly those in the Indian subcontinent, China and Southeast Asia, there is a deeply ingrained tradition of venerating older adults, with the care for aged parents being viewed as a sacred duty bestowed by the divine. Nevertheless, these societies are facing increasing pressure of globalization and westernization of care, reshaping conventional approaches to older adult care.

To steer the global response to aging, the WHO released the first World report on aging and health reviewing knowledge gaps and instigating a public health framework that identified four key areas: integrated care provision, long term care, age friendly environments and improved measurement and monitoring (3). Healthy aging was built on the notion of functional ability, seen as a combination of intrinsic capacity of individual, environmental characteristics, and interactions between individual and the environment. For the first time, a population aging metric reflecting both on longevity and health status was developed by the global burden of disease study conducted by Chang et al. (4). By incorporating 92 age related diseases, they extended our thinking beyond chronological age to account for both health status and disease severity, allowing cross country comparisons to inform policy decisions. Utilizing this metric, the top five countries globally with the highest aged-standardized disease burden rates are all in the Asia-Pacific region (Papua New Guinea, Marshall Islands, Vanuatu, Afghanistan, and Solomon Islands). Meanwhile Singapore, South Korea and Japan have some of the lowest age standardized disease burden. Comparing across countries, the equivalent age for the reference population (65 years) varied widely ranging from 76.1 years in Japan to 45.6 years in Papua New Guinea; meaning that a 76-year-old in Japan has the same age-related disease burden as a 65-year-old globally (4). The onus falls on the countries, and as mentioned in the WHO Global Strategy and Action Plan on Aging and Health (5), to accelerate action to ensure older adults can live a long and healthy life.

## Unique nature of Research Topic/issue and performance statistics

Our Research Topic was one of the first attempts to explore innovations in aged care and health service management across Asia and the Pacific, especially with a country- or region-specific approach. We adopted a co-design process of by working collaboratively with key country-level experts well-positioned to reach out to policymakers, academics, and researchers working in aged care and aged care management in different healthcare contexts in the region. The country co-leads or experts were well positioned to promote and disseminate findings to academic and policy networks. The issue and articles were widely marketed in the Asia-Pacific Region through networks, organizations, and media channels.

Since the launch of the Research Topic in July 2022, the issue has garnered significant interest among academics, researchers, and community, amassing close to 15,000 views. The published articles included in the issue have been viewed 12,663 times and downloaded 3,850 times. The level of engagement is also underscored by the 2,250 views on the topic itself. Moreover, the year 2023 has seen a continued upward trend in both views and downloads, indicating a rising interest on the subject matter and the research published therein (see Supplementary Figures 1, 2).

## Insights and advances from articles in the Research Topic

The Research Topic offers a rich and diverse collection of scholarly work on innovations in aged care and health service management across the Asia-Pacific region. We include a total of 14 articles, comprising 12 original research articles, one research report, and one perspective article. The research presented comes from nine countries, including Australia, Bangladesh, China, India, Japan, the Republic of Korea, Singapore, Thailand, and Vietnam. Table 1 presents a summary of studies included in the Research Topic, which are further described below.

**Table 1:** Summary of studies in the Research Topic.

**References**	**Title of article**	**Country**	**Type of article**	**Methods**	**Author keywords**
Boo and Oh	Perceptions of registered nurses on facilitators and barriers of implementing the AI-IoT-based healthcare pilot project for older adults during the COVID-19 pandemic in South Korea	Republic of Korea	Original Research	Qualitative	AI-IOT based healthcare; older adults; facilitators; barriers; digital literacy; public health
Dix et al.	Practical infection control training for Victoria's aged care workforce at the time of COVID-19 pandemic: a community case study	Australia	Original Research	Mixed methods	COVID19; education; health care professionals; infection control; personal protective equipment; program delivery; long term care; workplace safety
Hu et al.	Caring load and family caregivers' burden in China: the mediating effects of social support and social exclusion	China	Original Research	Quantitative	Psychological stress; family caregivers burden; older adults; social support; social exclusion; China
Katayama et al.	Relationship between tinnitus and olfactory dysfunction: audiovisual, olfactory and medical examinations	Japan	Original Research	Quantitative	Health checkup; sensory dysfunctions; olfactory test; dietary habits; smoking; alcohol
Kotani et al. ^*^	The research environment of critical care in three Asian countries: A cross-sectional questionnaire survey	Japan, Republic of Korea, Singapore	Original Research	Quantitative	Research activities; cross-sectional studies; community hospital; Asia; critical care
Lim et al.	Hospitalization and emergency department visits associated with potentially inappropriate medication in older adults: self-controlled case series analysis	Republic of Korea	Original Research	Quantitative	Potentially inappropriate medication; self-controlled case series; Poisson regression; older adult; pain medication; gastrointestinal medication; anticholinergics
Mackenzie et al.	The Vietnamese version of the Home Falls and Accidents Screening Tool (HOME FAST) - A preliminary study of validity and inter-rater reliability	Vietnam	Research Report	Mixed methods	Accidental falls; home hazards; assessment; cultural translation; validity expert panel
Mistry et al. ^*^	Stigma toward people with COVID-19 among Bangladeshi older adults	Bangladesh	Original Research	Quantitative	Dynamic ensemble selection; machine learning; middle aged patient; older patient; traumatic injury
Nhu et al.	Prediction of posttraumatic functional recovery in middle-aged and older patients through dynamic ensemble selection modeling	Taiwan, China	Original Research	Quantitative	Dynamic ensemble selection; machine learning; middle-aged patient; older patient; traumatic injury
Pardoel et al.	The implementation of community-based programs in Vietnam is promising in promoting health	Vietnam	Original Research	Mixed methods	Community based programs; community based health promotion; evaluation
Pengpid et al.	Bidirectional association between functional disability and multimorbidity among middle-aged and older adults in Thailand.	Thailand	Original Research	Quantitative	Multimorbidity; functional disability; longitudinal study; Thailand; bidirectional
Reddy et al. ^*^	Prevalence, Associated Factors, and Health Expenditures of Noncommunicable Disease Multimorbidity—Findings From Gorakhpur Health and Demographic Surveillance System	India	Original Research	Quantitative	Health and Demographic Surveillance; Health expenditure; India; multimorbidity; non communicable diseases
Sastry et al.	Toward Adapting the UN's Healthy Aging Agenda for India: Tailoring to Unique Historical Context and Traditions	India	Perspective	Not applicable	Older adult; healthy aging; healthcare; national health programs; digital health mission; India
Yi et al.	Does home and community-based services use reduce hospital utilization and hospital expenditure among disabled elders? Evidence from China	China	Original Research	Quantitative	Home and community based services; hospital utilization; hospital expenditure; substitution effect; health effect

^*^Kotani et al. was via Frontiers in Medicine submission; Mistry et al. and Reddy et al. was via General Section, Frontiers in Public Health submission.

Dix et al. present a case study on infection prevention and control (IPC) and the use of personal protective equipment (PPE) training for Australia's aged care workforce, set against the backdrop of the COVID-19 pandemic and involving residential aged care facilities (RACF) in the state of Victoria. Utilizing mixed methods techniques, the authors argue that the government, working in partnership with Monash University, an academic institution, had improved the design, development, and implementation of the program. This initiative is argued to be the largest state-funded program delivered to residential aged care workers and addresses the urgent need for rapid training of RACH staff in IPC and PPE. Over 4,200 RACF staff, including 1,207 facility champions, completed this program. This research sheds light on how collaborative efforts that integrate academic expertise can lead to swift and well-founded implementation, a strategy that was especially effective in the initial phase of the COVID-19 crisis in Australia.

A study from Bangladesh by Mistry et al. explored the COVID-19 related stigma among older adults in Bangladesh. Utilizing a cross-sectional design, over 1,000 older adults were surveyed through telephone interviews. The study identified that the prevalence of stigma related to COVID-19 was high among older adults, providing several implications to policymakers and public health personnel, and toward the design of mass media campaigns to inform and educate people on the stigma associated with COVID19. The authors also indicate that failure to understand the role of stigma might result in suboptimal reach of public health programs aimed at COVID19 prevention and management. Reflecting on this fascinating study, one could see similar pivots across the Asia-Pacific region, and relevance to many rural, remote, and disadvantaged communities.

Studies from China focused on community-based services, the role of informal carers, and long-term functional outcomes. Through a study utilizing China Health and Retirement Longitudinal Survey, Yi et al. conclude that the use of home- and community-based services can both reduce hospital utilization and expenditures among disabled older adults that contribute to their physical and psychological health. Hu et al. looked at informal care and examined the association between caring load and family caregivers' burden. The paper concludes that there is a significant positive association between caring load and caregiver burden and recommends more guidance services and support for family caregivers. Nhu et al. used a dynamic ensemble selection modeling approach in the prediction of posttraumatic functional recovery in middle-aged and older patients. They conclude that preexisting conditions can predict long-term functional outcomes, and thereby influence prognosis and clinical decision-making for older adult care.

Sastry et al. provided insights into the rich cultural heritage of India, and how the country is adapting the UN Health Aging Framework to the country's unique historical context and traditions. The paper highlights some interesting innovations by the Indian government such as “Savera Yojana” where older people can utilize digital technologies to reach friendly cops for personal security issues. Newer models of care such as 'adopting a granny' by school children, when a nursing home and school for orphans are cohabitated, are emerging in India, and seen as an avenue to improve intergenerational bonding. In another article by Reddy et al. the study examined the prevalence, associated factors and health expenditures of non-communicable disease multimorbidity from a health district in India. They identified that a significant proportion of the people had NCDs and argued that those with multimorbidity spent almost four times higher out of pocket expenditure than those without NCDs. The study suggests that national programs (such as Ayushman Bharat Scheme) can improve access to care and address the financial burden for care among older adults.

Japan, a pioneer in aged care solutions, presents valuable opportunities for international learning in this sector. The study by Katayama et al. examined the relationship between vision and hearing conditions among older adults. The study, which employs a mix of audiovisual, olfactory, and medical assessments, explores the connection between tinnitus and olfactory dysfunction. It determines that understanding the interplay between different sensory organs is crucial in assessing how sensory impairments affect cognitive function. The study provides some insights into the interconnectedness of sensory diseases and offers arguments into how and why integrated service provision needs to emerge in primary care.

A strong research environment is the backbone for evidence-informed policy and practice decision-making. In a cross-country study, Kotani et al. examined the critical care research environment in three Asian countries: Japan, South Korea, and Singapore. The study identified that having secured time for research activities, practicing at a university-affiliated hospital, and having clinical experience of 10 years or longer for whom? Were significantly associated with higher research productivity. The authors also argue the importance of building a collaborative environment between academia and non-academia (clinicians, public health professionals, community personnel etc.) as it contributes to the development of an evidence-based culture for care provision.

Boo and Oh, in their research on the use of Artificial Intelligence for a health care pilot project in South Korea, examined the perception of registered nurses on implementing smart technologies for older adults in primary care. The authors confirmed the need for future healthcare policies and strategies for the implementation of digital health technologies, particularly in the context of home visiting technologies. Also from South Korea, Lim et al. examined the risk of hospitalization and emergency department visits due to potentially inappropriate medication use among older adults. The study recommended the development of deprescribing strategies to control potentially inappropriate medication (PIM) and polypharmacy collectively.

A study conducted in Thailand by Pengpid et al. examined the bidirectional association between functional disability and multimorbidity among middle-aged and older adults. Using longitudinal data from the Thailand Health, Aging and Retirement Survey, the study identified that baseline multimorbidity increased the risk of functional disability, and further baseline functional disability increased the risk of incident multimorbidity. The authors argued that health services in Thailand should be reoriented to tailor the intervention to individuals with multimorbidity to prefer future functional disabilities.

The final two included studies from Vietnam by Pardoel et al. and Mackenzie et al. looked at the implementation of the intergenerational self-help clubs (ISHCs) and fall prevention tools respectively. Pardoel et al. conducted assessed the benefits of 97 ISHCs in nine provinces in Vietnam. The authors reported a high reach and uptake of services by ISHC, and a satisfaction rate of almost 75% among participants using them. Mackenzie et al. evaluated the validity and reliability of a home falls and accidents screening tool. Considering the relevance of home and informal care in Vietnam, the use of early screening and prevention tools cannot be underestimated.

## Future implications

A highly cited article published in Frontiers in Medicine by Franceschi et al. (6) argue that aging and age-related diseases share similar molecular and cellular mechanisms, and thus the primary focus of medicine should be on combating aging. The authors also venture to discuss on aging and rejuvenation (or feasible age extension) research and call for the importance of identifying biomarkers able to distinguish individuals with higher risk of developing age related diseases. From a public health perspective, this approach might seem unorthodox, but despite the progress achieved, it calls for wider engagement with a multidisciplinary audience. Our Research Topic, mainly from a public health and health management standpoint, shed light on a number of innovations on aged care in the Asia Pacific region. Although we don't argue that the challenges brought forth in this issue as comprehensive or even methodically organized, we recognize the importance to combat aging through innovative strategies that demand creative and unconventional thinking.

Our issue mainly makes the case for three arguments. First, aging is widely affecting the Asia-Pacific region, and countries are adopting innovative approaches and strategies to deal with aging. Second, Asia-Pacific countries bring rich cultural history and connotations to aged care, and its vital that traditional learnings and practices are nurtured to develop culturally sensitive and locally responsive models for aged care. Finally, the disease disparity and aged care practices across the region, provides an opportunity for cross-cultural and cross-country learnings, which can benefit both the developed and developing economics in the region and beyond.

## On reflection: summary of contributions

This Research Topic has featured original research articles (*n* = 12), short reports (*n* = 1) and perspectives (*n* = 1) from nine countries in the region (Australia, Bangladesh, China, India, Japan, Republic of Korea, Singapore, Thailand, and Vietnam). Commissioned in July 2022, we began with a unique process of involving country leads across the region in the promotion of the Research Topic and aimed at improving social impact through the rapid dissemination of research findings. Over the last 18 months, the Research Topic has generated 14,913 views and 3,850 article downloads. We included research on innovative models such as the intergenerational self-help clubs (Vietnam), artificial intelligence based smart care (Republic of Korea) and infection control training programs during COVID19 (Australia). The Research Topic also included studies that examined role of stigma toward older adults with COVID-19 (Bangladesh), cost benefits of home-based models (China) and the role of United Nations Healthy Aging agenda (India). The dominant themes emerging from the Research Topic mainly included: (i) service redesign, (ii) training for providers, (iii) shift to community/home-based care, and (iv) adapting successful global frameworks/instruments. Overall, it was particularly noteworthy to observe that several countries are thoughtfully assessing and evolving their unique and culturally rich traditional systems of older adult care to addressing contemporary needs and expectations.

## Author contributions

MB: Conceptualization, Writing – original draft, Writing – review & editing. AS: Conceptualization, Writing – review & editing. ZL: Conceptualization, Writing – review & editing.
