# Supplementary material for: Editorial: Innovations in older adult care and health service management: a focus on the Asia-Pacific region
Source: Front Public Health. 2024 Jan 31;12:1369827. doi: 10.3389/fpubh.2024.1369827 (PMC10865723; doi:10.3389/fpubh.2024.1369827)
Supplement: Supplementary file 1 [file Data_Sheet_1.docx]

**Supplementary Figure 1: Article performance statistics 2022-23, views and downloads**


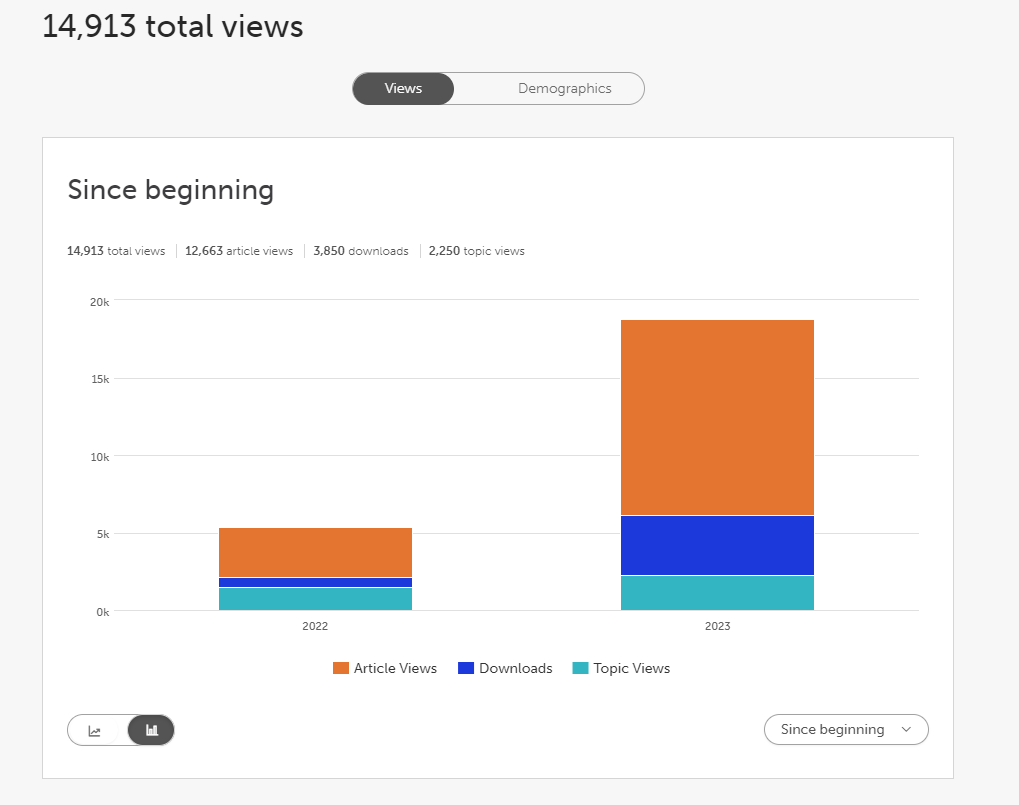


Note: The graph was generated on 15 Dec 2023

**Supplementary Figure 2: Article performance statistics 2023, months, views, and downloads**


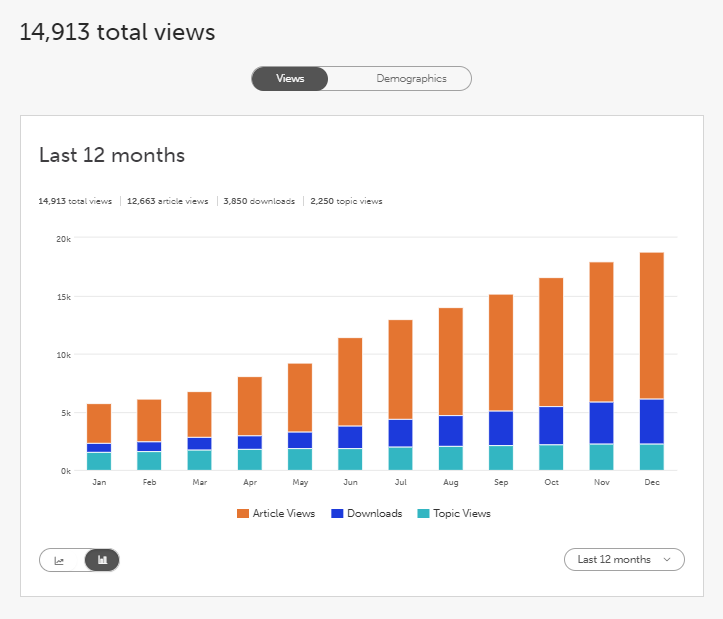


Note: The graph was generated on 15 Dec 2023

**Supplementary Table 1: Asia and the Pacific, sub-regions, and countries by income level**

| **Asia-Pacific Subregions** | **Low Income** | **Lower Middle Income** | **Upper Middle Income** | **High Income** |  |
| --- | --- | --- | --- | --- | --- |
| East and North-East Asia (ENEA) | Democratic People’s Republic of Korea (DPR Korea) | Mongolia | China | Japan; Republic of Korea |  |
| South-East Asia (SEA) |  | Cambodia; Indonesia; Lao People’s Democratic Republic (Lao PDR); Myanmar; Philippines; Timor-Leste; Viet Nam | Malaysia; Thailand | Brunei Darussalam; Singapore |  |
| South and South-West Asia (SSWA) | Afghanistan | Bangladesh; Bhutan; India; Iran (Islamic Republic of); Nepal; Pakistan; Sri Lanka | Maldives; Türkiye |  |  |
| North and Central Asia (NCA) |  | Kyrgyzstan; Tajikistan; Uzbekistan | Armenia; Azerbaijan; Georgia; Kazakhstan; Russian Federation; Turkmenistan |  |  |
| Pacific |  | Kiribati; Micronesia (F.S.); Papua New Guinea; Samoa; Solomon Islands; Vanuatu | American Samoa; Fiji; Marshall Islands; Palau; Tonga; Tuvalu | Australia; French Polynesia; Guam; Nauru; New Caledonia; New Zealand; Northern Mariana Islands |  |
|  |  |  |  |  |  |
| **Note:** Asia-Pacific subregion and country classification is based on the United Nations Economic and Social Commission for Asia and the Pacific categorisation. The income groupings are based on the World Bank 2022 Gross National Income, calculated using the World Bank Atlas Method: Low income ($1085 or less), lower-middle income ($1086 to $4255), upper middle income ($4256 to 13,205), and high income ($13,206 or more). | | | | |  |
|  |  |  |  |  |  |
|  |  |  |  |  |  |
|  |  |  |  |  |  |
